# Incidence and risk factors for amputation in Chinese patients with diabetic foot ulcers: a systematic review and meta-analysis

**DOI:** 10.3389/fendo.2024.1405301

**Published:** 2024-08-30

**Authors:** Yujie Zhang, Hui Liu, Yadi Yang, Chaochen Feng, Liwei Cui

**Affiliations:** ^1^ School of Humanities and Management, Zhejiang Chinese Medical University, Hangzhou, Zhejiang, China; ^2^ Department of Quality Management, Jining N0.1 People’s Hospital, Jining, Shandong, China

**Keywords:** amputation, diabetic foot ulcers, meta-analysis, risk factors, systematic review

## Abstract

**Objective:**

This study aimed to comprehensively analyze the incidence of amputation in Chinese patients with diabetic foot ulcers (DFUs).

**Methods:**

The Preferred Reporting Items for a Systematic Review and Meta-analysis (PRISMA) guidelines were used. The CNKI, Wanfang Data, VIP, PubMed, Web of Science, and Embase databases were searched to collect relevant literature on the incidence of amputation in Chinese patients with DFUs. Two researchers independently screened the literature, extracted data, and evaluated the risk of bias. The data were systematically analyzed using Stata 17.0 software to determine the incidence of amputation in this patient population.

**Results:**

A total of 25 papers were included in the study, revealing an incidence of amputation in Chinese patients with DFUs of 22.4% (95% confidence interval: 18.3–26.5%). The subgroup analysis revealed that a history of ulcers, Wagner grade >3, and diabetic peripheral vascular disease were the primary risk factors associated with a higher incidence of amputation in Chinese patients with DFUs (*P*<0.05). Among Chinese patients with DFUs, the amputation group and the non-amputation group showed significant differences in body mass index, duration of DFUs, total cholesterol, triglyceride, fasting blood glucose, white blood cell count, hemoglobin A1c, high-density lipoprotein cholesterol, low-density lipoprotein cholesterol, high-sensitivity C-reactive protein, and uric acid (*P*<0.05).

**Conclusion:**

The high incidence of amputation among Chinese patients with DFUs indicates that interventions should be implemented to prevent or minimize amputations.

**Systematic review registration:**

https://www.crd.york.ac.uk/prospero, identifier CRD42023463976.

## Introduction

Diabetes mellitus is a global public health threat that has increased in incidence over the last 20 years (1, 2). Currently, 537 million adults are affected by diabetes. Patients with diabetes are prone to several complications, among which diabetic foot ulcers (DFUs) are particularly prevalent (3). Each year, approximately 18.6 million people with diabetes experience foot ulcers worldwide (4). DFUs are characterized by infection or destruction of the soft tissues of the foot and are classified based on the affected area and extent of the lesions (5). They tend to heal poorly and require long-term, intensive treatment (6). DFUs are currently treated with a variety of therapies, including local surgical debridement (7), dressings to maintain a moist wound environment (8), wound offloading (9), vascular assessment (9), treatment of active infections, and glycemic control (10).

Studies have found the incidence rates of patients with a single occurrence of DFU in the United Kingdom, the United States, Spain, Denmark, and Australia to be 8.22% (11), 8.45% (12), 11.90% (13), 4.49% (14), and 5.35% (15), respectively. In Asia, the incidence rates of DFUs in Japan and Korea were 0.95% (16) and 4.90% (17), respectively. Meanwhile, a meta-analysis revealed that the incidence of recurrent DFUs among patients with diabetes was 38.01% (18). In China, the incidence of DFUs ranges from 17.03% to 42.84% (19–22), which is notably higher than in other countries.

A meta-analysis revealed that the prevalence of depression among patients with DFUs was 47%, with nearly half of the patients experiencing depressive symptoms (23). DFUs impose a heavy financial burden, with a direct cost of treatment estimated to be between 9 billion and 13 billion USD in the United States (24). Additionally, DFUs negatively impact patients’ quality of life (25). Notably, lower limb amputation is the most feared consequence of the disease (26). Given that DFUs significantly increase the risk of amputation, its adverse effects on individuals and society require urgent attention.

Worldwide, approximately 1.6 million people undergo amputations each year, of which approximately 33% are severe amputations. DFUs are the leading cause of nontraumatic amputations (4, 27), with more than 1 million diabetic patients undergoing nontraumatic lower extremity amputations each year, nearly 85% of which are due to DFUs (28). Crude estimates of 5-year mortality after amputation range from 39% to 68%, which is higher than the mortality rates of some common tumors (29). Therefore, it is crucial to implement effective interventions to prevent amputations in patients with DFUs.

A search of the Chinese and international literature revealed a lack of systematic reviews and meta-analysis studies on the incidence of amputation in Chinese patients with DFUs. Among three similar studies, two focused on the incidence and risk factors of lower limb amputation in patients with DFUs (30, 31), with only English-language studies being selected. Some differences were observed between the two studies, including variations in the reported incidence of combined lower extremity amputations. One of the studies lacked a quantitative analysis of the risk factors, while the other explored the epidemiology of diabetic foot amputation and its risk factors in the Middle East (31).

The incidence of amputation in Chinese patients with DFUs cannot be effectively characterized at present due to certain differences in the survey area, survey time, sample size, and other factors. Therefore, this systematic review and meta-analysis aimed to clarify the incidence of amputation in Chinese patients with DFUs and identify the factors influencing this incidence. This information can provide an evidence-based reference for early identification, diagnosis, and intervention to prevent amputation in these patients.

## Methods

### Protocol

The meta-analysis was conducted following the Preferred Reporting Items for Systematic Reviews and Meta-Analyses (PRISMA) guidelines (32). Please see the checklist in **Supplementary Material 8**. Our research protocol was registered with PROSPERO (CRD42023463976).

### Search strategy

The Chinese databases included in the study were CNKI, Wanfang Data, and VIP, while the English databases included were PubMed, Web of Science, and Embase. The search timeframe extended from the inception of the databases to December 2023. The search terms used were (“diabetic foot” OR “diabetic feet” OR “diabetic foot ulcer” OR “diabetic ulcer” OR “diabetic wound” OR “DF” OR “DFU”) AND (“amputation” OR “limb amputation” OR “limb loss”) AND (“China” OR “Chinese”). The researchers used subjects, article titles, or keywords to gather literature on the incidence of amputation in Chinese patients with DFUs and employed a literature tracing method to identify additional relevant literature.

### Study selection

The following inclusion criteria were adopted in the study. (1) Population: Chinese patients with DFUs. (2) Intervention and comparison: Whether patients with DFUs in China had undergone amputations. (3) Outcome: Accurate extraction of the incidence of amputation in Chinese patients with DFUs from the literature, or through indirect conversion based on the data in the text. (4) Study design: The study type was observational. We excluded the following studies: (1) reviews and conference abstracts, (2) repetitive publications or literature with data from the same study, and (3) literature with unavailable or untransformed data.

### Data extraction

Two researchers independently screened the literature, excerpted relevant information, and cross-checked it. The literature was screened twice—first by reading the title and abstract, and then by examining the full text—to ensure compliance with the inclusion and exclusion criteria, Relevant literature meeting all criteria was included. Specific data excerpts primarily included the following elements. (1) Basic characteristics: first author, survey time, survey area, average age of patients, etc. (2) Outcome indicators: incidence of amputation in Chinese patients with DFUs. If the amputation incidence rate was not specified, it was calculated using the formula: amputation incidence rate = (number of amputations/total sample size)×100. (3) Potential influencing factors: sex, smoking history, drinking history, hypertension, coronary artery disease, ulcer history, duration of DFUs, Wagner grade, neuropathy, peripheral vascular disease (PVD), retinopathy, nephropathy, age, duration of diabetes, body mass index (BMI), total cholesterol (TC), triglyceride (TG), fasting blood glucose (FBG), white blood cell (WBC) count, hemoglobin A1c (HbA1c), high-density lipoprotein cholesterol (HDL-C), low-density lipoprotein cholesterol (LDL-C), high-sensitivity C-reactive protein (hs-CRP), and uric acid (Ua).

### Quality assessment

The nine-point Newcastle–Ottawa Scale (NOS) (33) was used to assess the quality of cohort and case-control studies. This scale evaluates cohort studies based on eight entries, categorized into three major modules: selection, comparability, and exposure/outcome. The NOS evaluates the quality of the literature using the semi-quantitative principle of the star rating system. With the exception of comparability, which has a maximum two-star rating, the maximum rating for the entries is one star. A full score is 9 stars, with entries having a score of ≥7 being considered high-quality literature.

### Statistical analysis

The information from the included literature was entered into a database, and chi-square tests were conducted. Meta-analysis was performed using Stata 17.1 and RevMan 5.3 software. *I^2^* was used to test the heterogeneity of the included studies. If *I^2^* <50%, the fixed-effects model was used; if *I^2^* >50%, the random-effects model was used. If heterogeneity was detected, subgroup and sensitivity analyses were performed to further investigate its sources. Publication bias was assessed using a funnel plot, Egger’s test, and Begg’s test.

## Results

### Selection of studies and basic characteristics

**Figure 1:**
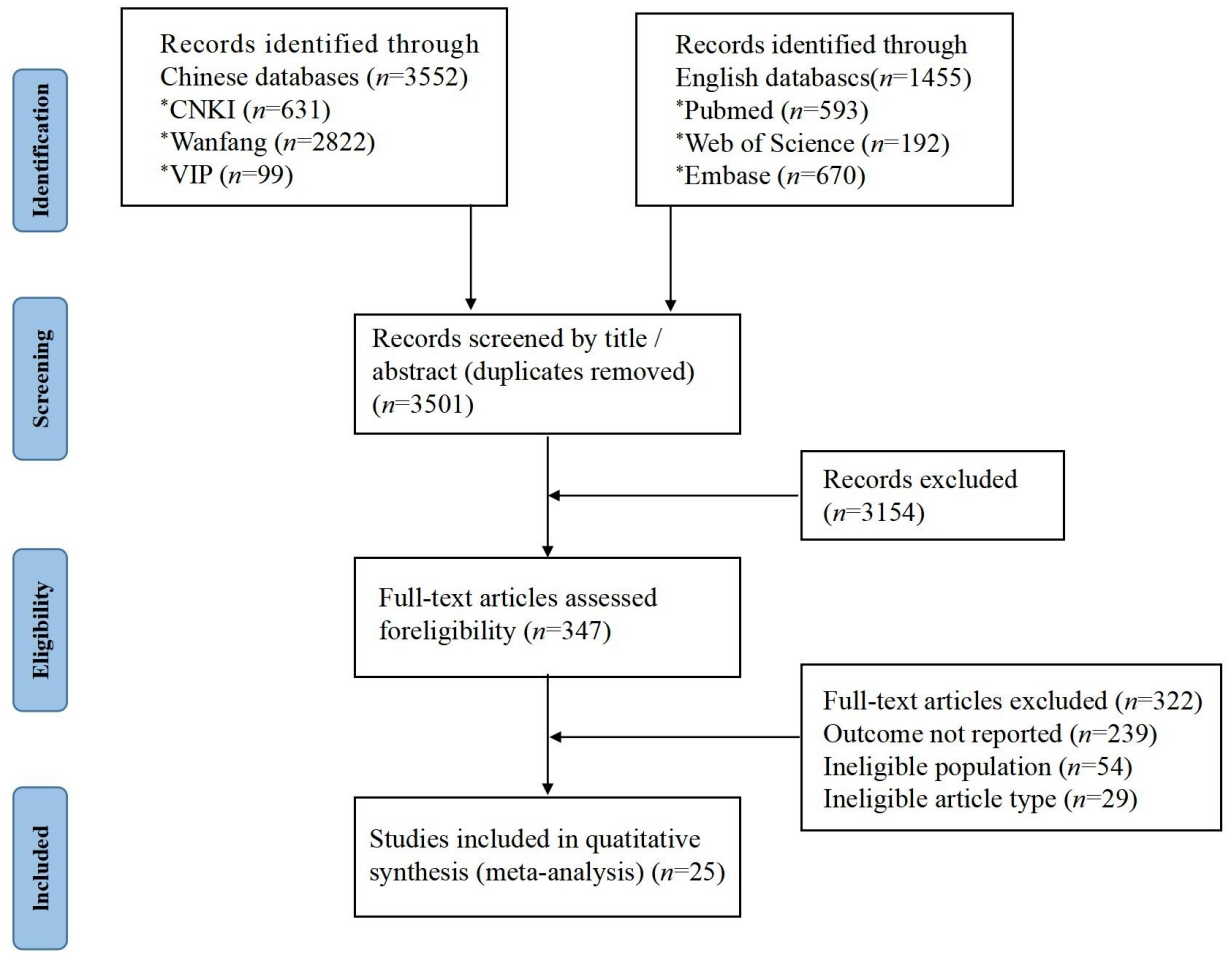
Flow chart of literature screening.

A total of 5,007 relevant articles were retrieved. After eliminating duplicates, the titles and abstracts were initially screened, followed by a full-text screening (**Figure 1**). The 25 included studies were all cohort studies published between 2000 and 2023. The effective sample size included 11,902 cases and 2,140 patients with DFUs who had experienced amputation. The incidence of amputation ranged from 5.10% to 57.03% (**Table 1**).

**Table 1:** Characteristics of the studies included in the meta-analysis.

Authors (Publication year)	Survey time	Provence	Type of research	Age (years)	Sample size (*n*)	Male/ Female (*n*)	Number of Incidence (*n*)	Incidence rate (%)	Quality score
Xiao(2009) (34)	2000.1-2008.11	Beijing	Cohort study	66.0 ± 10.4	436	274/162	97	22.25	7
Yan(2016) (35)	2014.1-2015.7	Shandong	Cohort study	—	150	89/61	24	26.00	9
Ye(2021) (36)	2015.12-2019.12	Anhui	Cohort study	63.3 ± 10.2	287	184/103	101	35.19	7
Tao(2020) (6)	2010.5-2017.9	Chongqing	Cohort study	66.2 ± 12.0	422	265/157	71	16.82	7
Wang(2014) (37)	2009.1-2011.1	Jiangsu	Cohort study	67.0 ± 12.3	194	110/84	12	6.19	8
Xie(2021) (38)	2009.1-2014.6	Chongqing	Cohort study	66.0	303	—	50	16.50	8
Gong(2023) (39)	2012.1-2020.12	Sichuan	Cohort study	65.1 ± 12.3	992	622/370	72	7.26	9
Peng(2022) (40)	2019.1-2022.3	Guizhou	Cohort study	58.1 ± 10.9	205	136/69	69	33.66	8
Guo(2019) (41)	2014.12-2018.9	Hunan	Cohort study	61.2 ± 12.0	475	294/181	59	12.42	7
Jiang(2015) (42)	2012.2-2013.1	—	Cohort study	64.0	669	435/201	133	19.88	9
Jiang(2015) (43)	2011.6-2013.5	—	Cohort study	—	196	—	10	5.10	9
Li(2011) (21)	2000.1-2009.9	Beijing	Cohort study	—	520	327/193	112	21.54	9
Lu(2020) (44)	2013.4-2020.7	Tianjin	Cohort study	66.7 ± 11.0	3654	2468/1186	363	9.93	9
Xu(2013) (45)	2011.1-2011.11	Shanghai	Cohort study	—	330	137/193	81	24.55	7
Shen(2020) (46)	2011.1-2015.12	Anhui	Cohort study	40-79	185	120/65	66	35.68	7
Mo(2018) (47)	2011.1-2015.12	Hainan	Cohort study	66.6 ± 9.80	189	118/71	31	16.40	9
Liu(2023) (48)	2015.10-2020.1	Jiangxi	Cohort study	64.9 ± 11.9	211	141/70	19	9.00	7
Zhu(2023) (49)	2013.7-2022.7	Zhejiang	Cohort study	62.1 ± 12.2	236	148/88	58	24.58	9
Sun(2016) (50)	2010.3-2012.8	Tianjin	Cohort study	—	375	268/107	113	30.13	7
Zhang(2023) (51)	2020.1-2021.10	Ningxia	Cohort study	—	390	208/182	138	35.38	7
Mei(2021) (52)	2019.7-12	Hubei	Cohort study	63.4 ± 9.8	156	106/50	24	15.38	8
Liu(2014) (53)	—	Shanghai	Cohort study	—	172	113/59	59	34.30	8
Wang(2007) (54)	2001.2-2006.11	Tianjin	Cohort study	68 ± 9	249	145/104	142	57.03	9
Cheng(2020) (55)	2014.3-2018.10	Beijing	Cohort study	—	573	384/189	74	12.91	8
He(2017) (56)	2009.4-2012.3	Shanghai	Cohort study	—	333	—	162	48.65	7

The NOS scores of the included studies ranged from 7 to 9, indicating high quality. However, most studies lacked follow-up time and completeness of follow-up (**Supplementary Material 1**).

### Incidence of amputation in Chinese patients with DFUs

**Figure 2:**
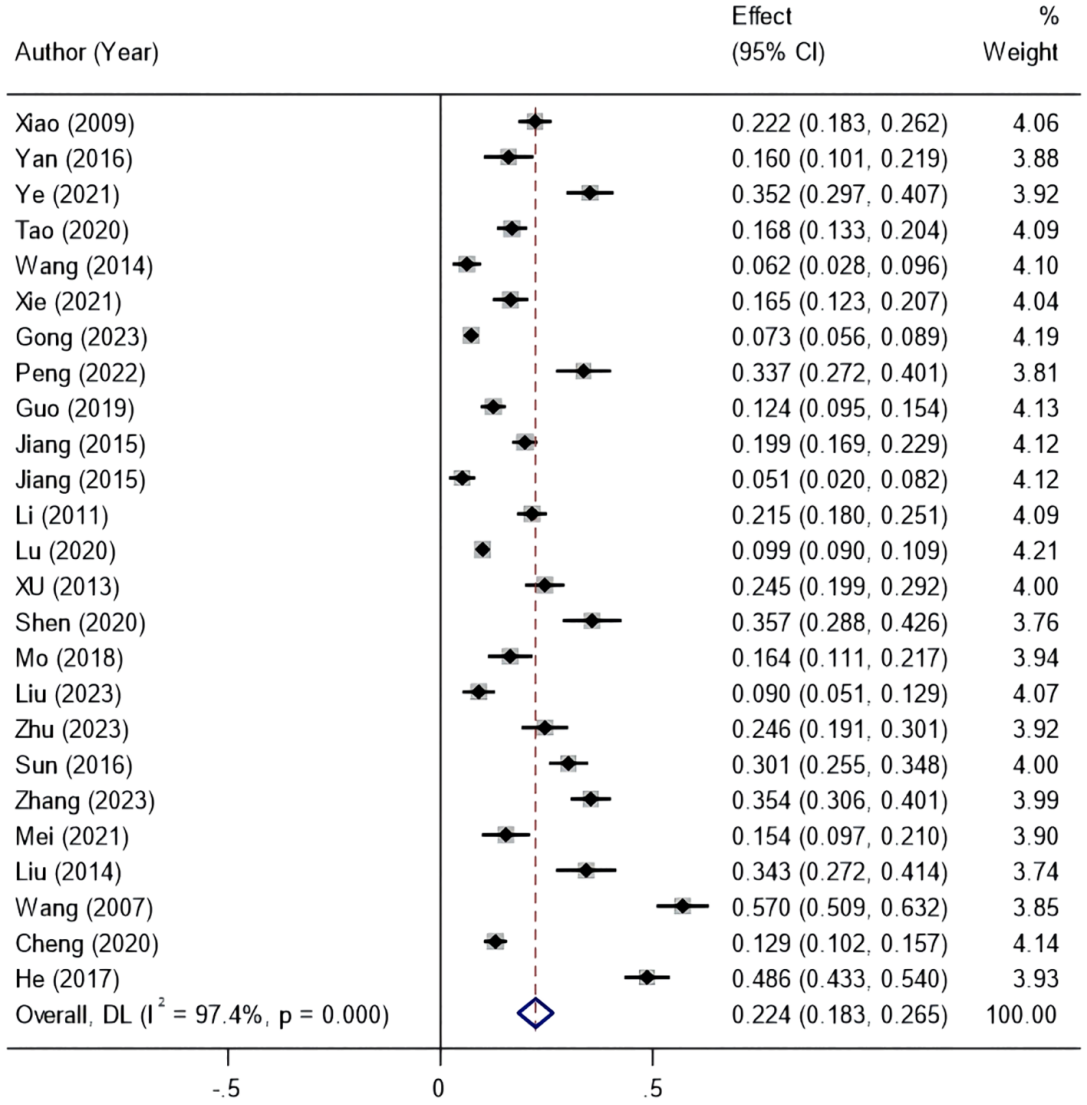
Forest plot of the incidence of amputation in patients with diabetic foot ulcers based on the random-effects model.

The heterogeneity test for the included studies showed *I^2 =^* 97.4% (*P*<0.01), so the random-effects model was chosen for the meta-analysis. The results showed that the incidence of amputation in Chinese patients with DFUs was 22.4% (95% *CI* 18.3–26.5%) (**Figure 2**).

### Publication bias

**Figure 3:**
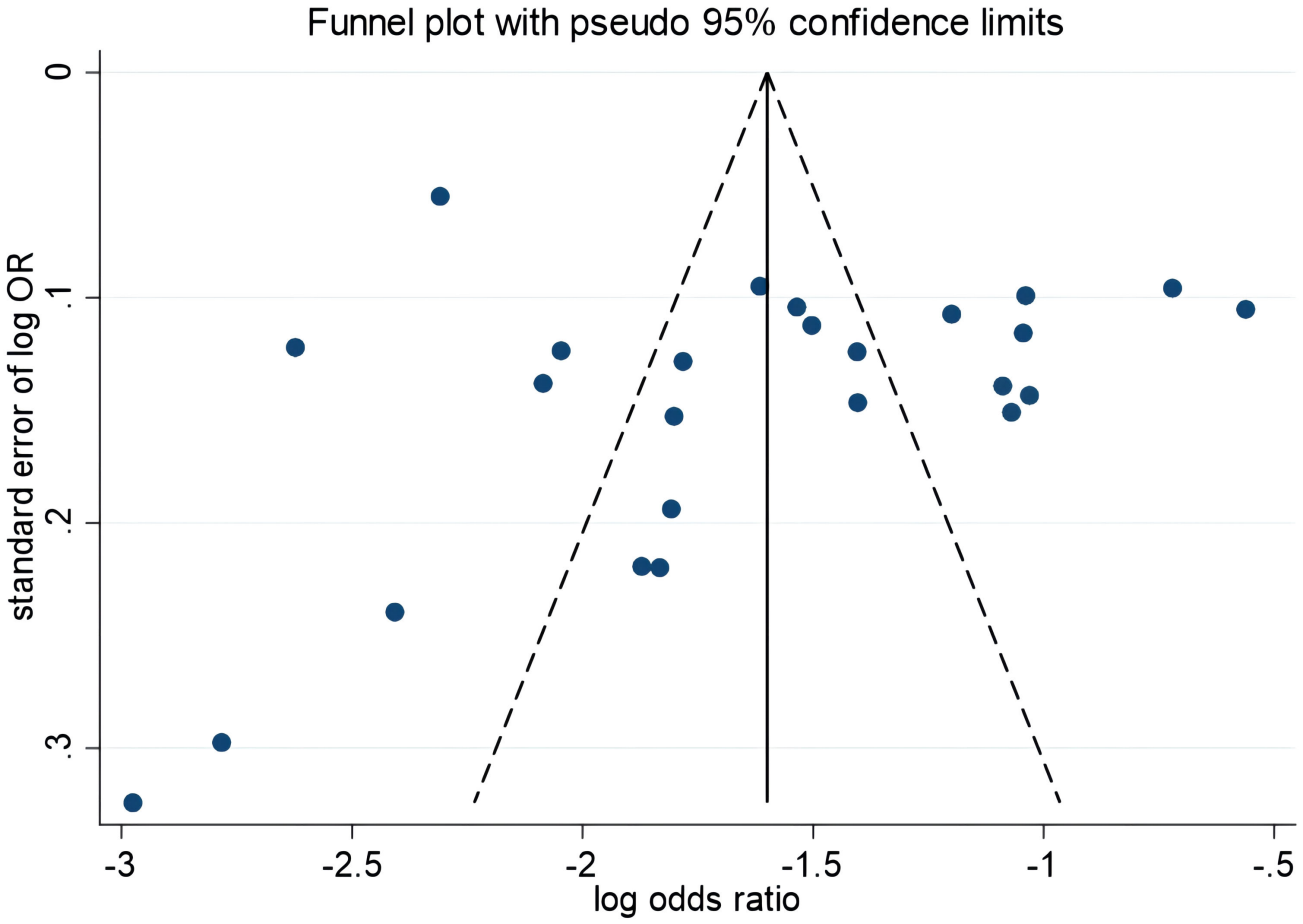
Funnel plot.

The funnel plot results showed symmetry in the graphical distribution on both sides (**Figure 3**). Egger’s and Begg’s tests were used to evaluate publication bias. *P*>0.05 indicated no significant publication bias. Egger’s test (*P*=0.792) and Begg’s test (*P*=0.059) suggested that the possibility of publication bias was small.

### Subgroup analysis

The heterogeneity test results showed that the literature mentioning hypertension, ulcer history, Wagner grade, age, duration of DFUs, TG, WBC, and HbA1c was highly heterogeneous (*I^2^* >50%), so a random-effects model was chosen. Sex, smoking history, drinking history, coronary artery disease, neuropathy, PVD, retinopathy, nephropathy, BMI, duration of diabetes, TC, FBG, HDL-C, LDL-C, hs-CRP, and Ua factors were more homogeneous (*I^2^* <50%); thus, a fixed-effects model was applied.

Subgroup analysis showed that the incidence of amputation was higher among Chinese patients with DFUs who had an ulcer history, Wagner grade >3, and diabetic peripheral vascular disease (*P*<0.05) (**Table 2**) (**Supplementary Material 3**). Statistically significant differences were found between the amputation and non-amputation groups of Chinese patients with DFUs in terms of BMI, duration of DFUs, TC, TG, FBG, WBC, HbA1c, HDL-C, LDL-C, hs-CRP, and Ua (*P*<0.05) (**Table 3**) (**Supplementary Material 4-7**).

**Table 2:** Incidence rate of amputation in patients with diabetic foot ulcers.

Subgroups	Experiment group	Control group	No. of studies	Heterogeneity		Effect model	*OR (*95%*CI)*	*Z*	*P*
				*I^2^* (%)	*P*				
Gender	Male	Female	7	0	0.92	Fixed	1.11 (0.91, 1.35)	0.99	0.32
Smoking history	Yes	No	5	0	0.63	Fixed	1.04 (0.84, 1.29)	0.40	0.69
Drinking history	Yes	No	3	0	0.43	Fixed	0.77 (0.60, 1.01)	1.90	0.06
Coronary artery disease	Yes	No	3	0	0.80	Fixed	0.78 (0.56, 1.09)	1.46	0.15
Hypertension	Yes	No	5	69	0.01	Random	0.94 (0.59, 1.51)	0.25	0.80
Ulcer history	Yes	No	3	69	0.04	Random	2.28 (1.38, 3.76)	3.22	0.001
Wanger grade	≤3	>3	5	82	<0.001	Random	0.21 (0.09, 0.47)	3.80	<0.001
Neuropathy	Yes	No	6	48	0.09	Fixed	0.96 (0.73, 1.28)	0.25	0.80
PVD	Yes	No	4	0	0.40	Fixed	1.66 (1.28, 2.15)	3.83	<0.001
Retinopathy	Yes	No	5	4	0.38	Fixed	0.89 (0.70, 1.12)	1.01	0.31
Nephropathy	Yes	No	4	0	0.55	Fixed	1.12 (0.86, 1.45)	0.83	0.41

**Table 3:** Comparison of characteristics among the non-amputation and amputation groups.

Subgroups	No. of studies	Heterogeneity		Effect model	*MD (*95%*CI)*	*Z*	*P*
		*I^2^* (%)	*P*				
Age	7	81	<0.001	Random	-1.49 (-3.56, 0.58)	1.41	0.16
BMI	4	29	0.24	Fixed	-0.71 (-0.89, -0.53)	7.74	<0.001
Duration of diabetes (Year)	5	35	0.19	Fixed	0.05 (-0.40, 0.50)	0.21	0.84
Duration of DFUs (months)	3	97	<0.001	Random	-2.32 (-3.61, -1.03)	3.52	<0.001
TC (mmol/L)	5	33	0.20	Fixed	0.41 (0.28, 0.53)	6.15	<0.001
TG (mmol/L)	7	75	<0.001	Random	0.20 (0.06, 0.34)	2.86	0.004
FBG	4	0	0.57	Fixed	-0.44 (-0.65, -0.23)	4.11	<0.001
WBC (10^9/L)	7	57	0.03	Random	-2.34 (-3.02, -1.65)	6.64	<0.001
HbA1c (g/L)	6	58	0.04	Random	-0.48 (-0.87, -0.09)	2.43	0.02
HDL-C (mmol/L)	5	38	0.17	Fixed	0.14 (0.11, 0.17)	9.08	<0.001
LDL-C (mmol/L)	6	0	0.88	Fixed	0.23 (0.17, 0.29)	7.91	<0.001
hs-CRP (mmol/L)	4	0	0.97	Fixed	-3.37 (-4.15, -2.59)	8.45	<0.001
Ua (μmol/L)	4	31	0.23	Fixed	20.70 (15.58, 25.83)	7.92	<0.001

### Sensitivity analysis

Sensitivity analyses were performed using the article-by-article exclusion method. The variables for coronary heart disease and BMI showed considerable changes in heterogeneity after excluding Ye’s study (36). Similarly, the variables for diabetes duration and HDL-C showed substantial changes in heterogeneity after excluding Wang’s (37) and Jiang’s (43) studies, respectively. This suggests that these studies may be sources of heterogeneity, leading to their exclusion.

After these exclusions, the sensitivity analysis was performed again with the adjusted analytical model. The *OR* or *MD* values and 95% *CI* from the two analysis models for each subgroup variable were similar (**Supplementary Material 2**), indicating that the sensitivity of this study was low and the results were more stable.

## Discussion

Our meta-analysis showed that the incidence of amputation in patients with DFUs was 22%. In comparison, a meta-analysis showed that the incidence of amputation in patients with DFUs was 19% (57). However, a meta-analysis of Middle Eastern countries found that the incidence of amputation in patients with DFUs was 33% (31). China’s developing economy and vigorous promotion of medical science popularization has led to improved health literacy and increased health awareness. Disease awareness has been increasing among patients with DFUs, and they have gained understanding of potential health hazards such as amputation. However, the incidence of amputation among patients with DFUs in China remains high overall.

China currently lacks medical institutions for diabetes, and hospitals lack specialized departments and clinicians for DFUs. This results in a shortage of medical resources that prevents residents from accessing timely and effective diagnosis and treatment for foot ulcers (58). While patients’ awareness of diabetic foot disease has increased, significant misconceptions still remain (59). Some patients recognize their condition, but delay seeking consultation because of their low economic status. For example, alcohol and trauma stickers are sometimes used to self-treat wounds without proper medical guidance, thus missing the optimal time for treatment, and eventually leading to lower limb amputation (60, 61).

The incidence of amputation is higher among patients with DFUs, who have a ulcer and Wagner >3 grade (*P*<0.05). A significant difference was also observed in the duration of diabetic foot disease between the amputation and non-amputation groups (*P*<0.05). This is consistent with the studies by Jiang (43) and Gong (39). All three indicators reflect the severity of the disease, with amputation rates typically increasing with disease severity. Foot ulcers with Wagner grade 1 to 2 generally heal because they do not involve bone tissue. However, a Wagner grade of 3 or higher indicates that the infection involves bone, and the amputation rate is 11 times higher (62).

Our results showed a significant difference in BMI between the amputation and non-amputation groups (*P*<0.05). BMI is influenced by a patient’s lifestyle, and a high BMI may reflect deficiencies in disease awareness. This highlights the importance of improving patients’ disease-related knowledge and awareness. WBC and hs-CRP were significantly higher in the amputation group compared to the non-amputation group (*P*<0.05), consistent with findings from Xie’s study (35). High WBC and hs-CRP levels in patients in the amputation group suggest a persistent and severe infection in diabetic foot patients, which correlates with a poor prognosis (34).

Significant differences were observed between the amputation and non-amputation groups in terms of TC, TG, HDL-C, and LDL-C (*P*<0.05). Nutritional indicators are important for determining the effect and prognosis of DFU treatment, as they are essential for evaluating the nutritional status of patients. Li’s study (21) showed that these indicators are important factors in preventing amputation. LDL-C can cause malnutrition and increased mortality, while HDL-C has anti-atherosclerotic properties (61, 63). Together, these factors can exacerbate the patient’s nutritional status and affect the healing of foot ulcers. This highlights the need for enhanced nutritional support therapy for patients with DFUs.

Studies have demonstrated that intensive glycemic control can reduce the risk of amputation among patients with DFUs (64, 65). Our results showed that FBG and HbA1c levels were significantly higher among patients in the amputation group (*P*<0.05). A meta-analysis of randomized controlled trials demonstrated that intensive glycemic control reduces the risk of amputation by 35% in patients with diabetic foot syndrome (66). This suggests that poor glycemic control is associated with a higher likelihood of healing difficulties and an increased risk of amputation.

## Limitations

This study has some limitations. First, the meta-analysis primarily included cross-sectional single-arm studies, where respondents were susceptible to subjective factors, leading to significant heterogeneity between studies. Second, the limited number of included studies, and the fact that the minimum number of studies included in each subgroup analysis was three, might have impacted the results. Third, relatively few studies were from the central and western regions of China. Fourth, our study was restricted to publications in Chinese and English. This implies that important local studies published in journals in other languages may have been overlooked, which could have led to bias in our findings. Therefore, the primary scope of the investigation was limited to certain provinces and cities, which might have affected the comprehensiveness and representativeness of the results.

## Conclusions

We systematically evaluated the incidence of amputation and associated risk factors through meta-analysis. We found a high incidence of amputation, with ulcer history, BMI, TC, TG, FBG, leukocytes, and glycosylated hemoglobin being important factors. Therefore, timely and appropriate interventions for these patients are necessary. We must actively encourage patients to adopt healthier lifestyles and improve their health literacy. Additionally, effectively control of blood glucose levels and infections, improvement in blood supply to the lower limbs, and provision of nutritional support are crucial to effectively reduce the incidence of amputation among Chinese patients with DFUs.

## Data Availability

The raw data supporting the conclusions of this article will be made available by the authors, without undue reservation.
