# Supplementary material for: Incidence and risk factors for amputation in Chinese patients with diabetic foot ulcers: a systematic review and meta-analysis
Source: Front Endocrinol (Lausanne). 2024 Aug 30;15:1405301. doi: 10.3389/fendo.2024.1405301 (PMC11393406; doi:10.3389/fendo.2024.1405301)
Supplement: Supplementary file 1 [file DataSheet1.doc]

**Supplementary Materials**

**Supplementary material 1. Results of bias risk assessment of included studies.**

| Authors | Selection of population | | | | Comparability | Evaluation of exposure or outcome | | | NOS  score |
| --- | --- | --- | --- | --- | --- | --- | --- | --- | --- |
| Xiao(2009)^[1]^ | 1 | 1 | 1 | 1 | 2 | 1 | 0 | 0 | 7 |
| Yan(2016)^[2]^ | 1 | 1 | 1 | 1 | 2 | 1 | 0 | 0 | 7 |
| Ye(2021)^[3]^ | 1 | 1 | 1 | 1 | 2 | 1 | 0 | 0 | 7 |
| Tao(2020)^[4]^ | 1 | 1 | 1 | 1 | 2 | 1 | 1 | 1 | 9 |
| Wang(2014)^[5]^ | 1 | 1 | 1 | 1 | 2 | 1 | 1 | 0 | 8 |
| Xie(2021)^[6]^ | 1 | 1 | 1 | 1 | 2 | 1 | 0 | 0 | 7 |
| Gong(2023)^[7]^ | 1 | 1 | 1 | 0 | 2 | 1 | 0 | 0 | 6 |
| Peng(2022)^[8]^ | 1 | 1 | 1 | 1 | 2 | 1 | 0 | 0 | 7 |
| Guo(2019)^[9]^ | 1 | 1 | 1 | 1 | 2 | 1 | 0 | 0 | 7 |
| Jiang(2015)^[10]^ | 1 | 1 | 1 | 1 | 2 | 1 | 0 | 0 | 7 |
| Jiang(2015)^[11]^ | 1 | 1 | 1 | 1 | 2 | 1 | 1 | 1 | 9 |
| Li(2011)^[12]^ | 1 | 1 | 1 | 1 | 2 | 1 | 0 | 0 | 7 |
| Lu(2020)^[13]^ | 1 | 1 | 1 | 1 | 2 | 1 | 0 | 0 | 7 |
| Xu(2013)^[14]^ | 1 | 1 | 1 | 1 | 2 | 1 | 1 | 0 | 8 |
| Shen(2020)^[15]^ | 1 | 1 | 1 | 1 | 2 | 1 | 1 | 0 | 8 |
| Mo(2018)^[16]^ | 1 | 1 | 1 | 1 | 2 | 1 | 1 | 1 | 9 |
| Liu(2023)^[17]^ | 1 | 1 | 1 | 0 | 2 | 1 | 1 | 1 | 8 |
| Zhu(2023)^[18]^ | 1 | 1 | 1 | 1 | 2 | 1 | 0 | 0 | 7 |
| Sun(2016)^[19]^ | 1 | 1 | 1 | 1 | 2 | 1 | 1 | 1 | 9 |
| Zhang(2023)^[20]^ | 1 | 1 | 1 | 1 | 2 | 1 | 1 | 1 | 9 |
| Mei(2021)^[21]^ | 1 | 1 | 1 | 1 | 2 | 1 | 1 | 1 | 9 |
| Liu(2014)^[22]^ | 1 | 1 | 1 | 1 | 2 | 1 | 1 | 1 | 9 |
| Wang(2007)^[23]^ | 1 | 1 | 1 | 1 | 2 | 1 | 0 | 0 | 7 |
| Cheng(2020)^[24]^ | 1 | 1 | 1 | 1 | 2 | 1 | 0 | 0 | 7 |
| He(2017)^[25]^ | 1 | 1 | 1 | 1 | 2 | 1 | 1 | 1 | 9 |

**Additional References***

Xiao T, Wang AH, Xu ZR, Wang YZ, Jiang YF, Gu HB, et al. Analysis of amputation risk factors in 436 patients with diabetic foot ulcers. Chin J Endocrinol Metab. (2009) 25:591-594. doi:10.3760/cma.j.issn.1000-6699.2009.06.002 [In Chinese]

Yan L, Ma CH, Zhang JX, Zhang MH, Wang ZP. Logistic regression analysis of risk factors of amputation among inpatients with diabetic foot and intervention. Chin J Nosocomiol. (2016) 26(04):824-826. doi:10.11816/cn. ni. 2016-152341 [In Chinese]

Ye L, Deng JX, Cao DS, Xie J, Pan FM. Risk factors for amputation in patients with diabetic foot ulcer. Acta Universitatis Medicinalis Anhui. (2021) 56(04):608-612. doi: 10.19405/j.cnki. issn1000-1492.2021.04.020 [In Chinese]

Tong T, Yang C, Tian W, Liu ZP, Liu B, Cheng J, et al. Phenotypes and outcomes in middle-aged patients with diabetic foot ulcers: a retrospective cohort study. J Foot Ankle Res. (2020) 13(1):24. doi:10.1186/s13047-020-00386-z

Wang A, Sun X, Wang W, Jiang K. A study of prognostic factors in Chinese patients with diabetic foot ulcers. Diabet Foot Ankle. (2014) 5:10.3402/dfa.v5.22936. doi:10.3402/dfa.v5.22936

Xie P, Deng B, Zhang X, Li YY, Du CZ, Rui SL, et al. Time in range in relation to amputation and all-cause mortality in hospitalised patients with diabetic foot ulcers. Diabetes Metab Res Rev. (2022) 38(2):e3498. doi:10.1002/dmrr.3498

Gong H, Ren Y, Li Z, Zha PP, Bista R, Li Y, et al. Clinical characteristics and risk factors of lower extremity amputation in the diabetic inpatients with foot ulcers. Front Endocrinol (Lausanne). (2023) 14:1144806. doi:10.3389/fendo.2023.1144806

Peng X, Gou D, Zhang L, Wu HM, Chen Y, Shao X, et al. Status and influencing factors of lower limb amputation in patients with diabetic foot ulcer. Int Wound J. (2023) 20(6):2075-2081. doi:10.1111/iwj.14076

Guo Z, Yue C, Qian Q, He H, Mo Z. Factors associated with lower-extremity amputation in patients with diabetic foot ulcers in a Chinese tertiary care hospital. Int Wound J. (2019) 16(6):1304-1313. doi:10.1111/iwj.13190

Jiang Y, Ran X, Jia L, Yang C, Wang P, Ma J, et al. Epidemiology of type 2 diabetic foot problems and predictive factors for amputation in China. Int J Low Extrem Wounds. (2015) 14(1):19-27. doi:10.1177/1534734614564867

Jiang Y, Wang X, Xia L, Fu X, Xu Z, Ran X, et al. A cohort study of diabetic patients and diabetic foot ulceration patients in China. Wound Repair Regen. (2015) 23(2):222-230. doi:10.1111/wrr.12263

Li X, Xiao T, Wang Y, Gu H, Liu Z, Jiang Y, et al. Incidence, risk factors for amputation among patients with diabetic foot ulcer in a Chinese tertiary hospital. Diabetes Res Clin Pract. (2011) 93(1):26-30. doi:10.1016/j.diabres.2011.03.014

Lu Q, Wang J, Wei X, Wang G, Xu Y, Lu Z, Liu P. Cost of diabetic foot ulcer management in China: A 7-year single-center retrospective review. Diabetes Metab Syndr Obes. (2020) 13:4249-4260. doi:10.2147/DMSO.S275814

Xu L, Qian H, Gu J, Shi J, Gu X, Tang Z. Heart failure in hospitalized patients with diabetic foot ulcers: clinical characteristics and their relationship with prognosis. J Diabetes. (2013) 5(4):429-438. doi:10.1111/1753-0407.12062

Shen JF, Jiang RM, Wang ZQ, Li M, Li J, Xie SY, Kang JJ. Recurrence and influencing factors of diabetic foot ulcer in patients with type 2 diabetes mellitus. Chin J Burns. (2020) 36(10):947-952. doi:10.3760/cma.j.cn501120-20190726-00315 [In Chinese]

Mo ZW, Chen DX, Gao YY, Quan HB, Chen CR, Wang F, Zhang HC.. Risk factors for foot ulcer recurrence in diabetic patients with new-onset foot ulcers. Chin Trop Med. (2018) 18(07):716-719. doi:10.13604/j.cnki.46-1064/r.2018.07.21 [In Chinese]

Liu WJ. Analysis of factors influencing recurrent diabetic foot ulcers. MA thesis. Nanchang University. (2023). doi:10.27232/d.cnki.gnchu.2023.000720 [In Chinese]

Zhu JJ, Li SM. Study on limb infection in diabetic patients and risk factor analysis of amputation. Chin J Health Lab Tec. (2023) 33(15):1892-1896. [In Chinese]

Sun XX, Wang PH, Zhang JH, Hou XL, Zhao RY, Li XW. Could antibiotics be stopped after symptoms disappear in patients with diabetic foot infection? Chin J Diabetes Mellitus. (2016) 8(6):346-350. doi:10.3760/cma.j.issn.1674-5809.2016.06.007 [In Chinese]

Zhang J, Li HF, Li XM, Yao M, Ma HZ, Ma Q. Construction of recurrence risk prediction model for diabetic foot ulcer on the basis of Logistic regression, support vector machine and BP neural network model. Chin Gen Pract. (2023) 26(32):4013-4019. [In Chinese]

Mei T. Study on the status and influencing factors of ulcer-free survival days in diabetic foot ulcer patients. MA thesis. Huazhong University of Science and Technology. (2021). doi:10.27157/d.cnki.ghzku.2021.001910 [In Chinese]

Liu CB, Huang Y, Ye JN, Ni PW, Lu SL. Prognostic factors for diabetic foot ulcer. Chin J [Trauma](https://navi.cnki.net/knavi/pubdetail?pubtype=journals&baseid=ZHCS). (2014) 30(04):291-297. doi:10.3760/cma.j.issn.1001-8050.2014.04.002 [In Chinese]

Wang PH, Yu DM, Chu YJ, Song ZQ, Wang JC, Xu J, et al. Research on the clinical features and effective factors of 249 diabetic patients with deep foot infection. Chin J Med. (2007) 87(26):1828-1831. [In Chinese]

Cheng Y, Zu P, Zhao J, Shi L, Shi H, Zhang M, Wang A. Differences in initial versus recurrent diabetic foot ulcers at a specialized tertiary diabetic foot care center in China. J Int Med Res. (2021) 49(1):300060520987398. doi:10.1177/0300060520987398

He Y, Qian H, Xu L, Zhang S, Gu X, Gu J, et al. Association between estimated glomerular filtration rate and outcomes in patients with diabetic foot ulcers: a 3-year follow-up study. Eur J Endocrinol. (2017) 177(01):41-50. doi:10.1530/EJE-17-0070

**Supplementary material 2. Sensitivity analysis.**

| Subgroups | Random Model | | Fixed Model | |
| --- | --- | --- | --- | --- |
|  | *0R*/*MD* | 95%*CI* | *0R*/*MD* | 95%*CI* |
| Gender | 1.10 | (0.91, 1.35) | 1.11 | (0.91, 1.35) |
| Smoking history | 1.05 | (0.84, 1.30) | 1.04 | (0.84, 1.29) |
| Drinking history | 0.78 | (0.60, 1.01) | 0.77 | (0.60, 1.01) |
| Coronary artery disease | 0.79 | (0.56, 1.09) | 0.78 | (0.56, 1.09) |
| Hypertension | 0.94 | (0.59, 1.51) | 1.00 | (0.78, 1.28) |
| Ulcer history | 2.28 | (1.38, 3.76) | 2.28 | (1.74, 2.99) |
| Wanger grade | 0.21 | (0.09, 0.47) | 0.25 | (0.19, 0.33) |
| Neuropathy | 0.99 | (0.63, 1.55) | 0.96 | (0.73, 1.28) |
| PVD | 1.62 | (1.25, 2.11) | 1.66 | (1.28, 2.15) |
| Retinopathy | 0.89 | (0.70, 1.13) | 0.89 | (0.70, 1.12) |
| Nephropathy | 1.11 | (0.86, 1.45) | 1.12 | (0.86, 1.45) |
| Age | -1.49 | (-3.56, 0.58) | 0.16 | (-0.41, 0.73) |
| BMI | -0.54 | (-1.00, -0.08) | -0.71 | (-0.89, -0.53) |
| Duration of diabetes (Year) | 0.23 | (-0.47, 0.92) | 0.05 | (-0.40, 0.50) |
| Duration of DFUs (months) | -2.32 | (-3.61, -1.03) | -0.98 | (-1.11, -0.84) |
| TC (mmol/L) | 0.38 | (0.22, 0.55) | 0.41 | (0.28, 0.53) |
| TG (mmol/L) | 0.20 | (0.06, 0.34) | 0.17 | (0.13, 0.20) |
| FBG | -0.44 | (-0.65, -0.23) | -0.44 | (-0.65, -0.23) |
| WBC (10^9/L) | -2.34 | (-3.02, -1.65) | -1.73 | (-1.98, -1.48) |
| Hb (g/L) | 10.17 | (6.56, 13.78) | 10.03 | (6.92, 13.13) |
| HbA1c (g/L) | -0.48 | (-0.87, -0.09) | -0.31 | (-0.43, -0.18) |
| HDL-C (mmol/L) | 0.13 | (0.09, 0.18) | 0.14 | (0.11, 0.17) |
| LDL-C (mmol/L) | 0.23 | (0.17, 0.29) | 0.23 | (0.17, 0.29) |
| hs-CRP (mmol/L) | -3.37 | (-4.15, -2.59) | -3.37 | (-4.15, -2.59) |
| Ua (μmol/L) | 23.88 | (11.85, 35.91) | 20.70 | (15.58, 25.83) |

Abbreviations: PVD, peripheral vascular disease; BMI, body mass index; DFUs, diabetic foot ulcers; TC, total cholesterol; TG, triglyceride; FBG, fasting blood glucose; WBC, white blood cell; Hb, hemoglobin; HbA1c, haemoglobin A1c; HDL-C, high density lipoprotein cholesterol; LDL-C, low density lipoprotein cholesterol; hs-CRP, hyper-sensitive C reactive protein; Ua, uric acid.

**A**


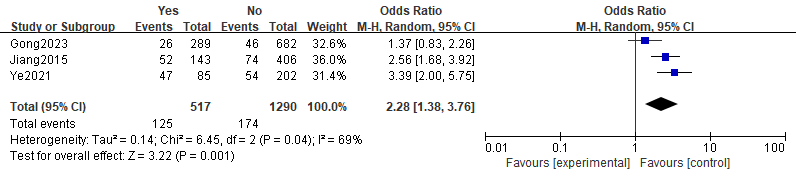


**B**


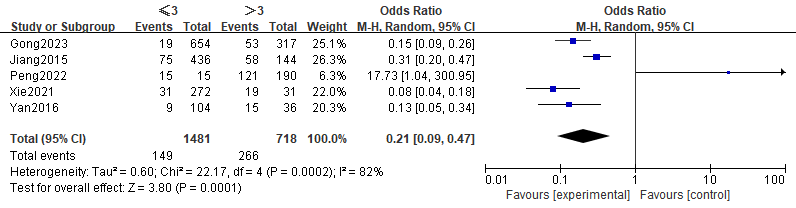


**C**


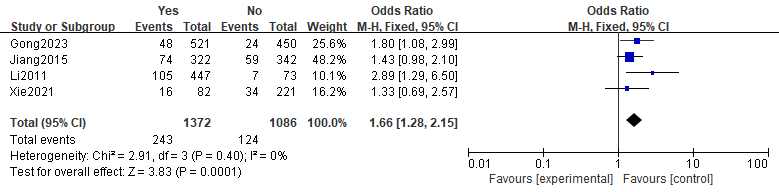


**Supplementary material 3**. Meta-analysis results of the incidence for amputation in Chinese patients with diabetic foot ulcers in the two groups. (A) Ulcer history; (B) Wanger grade; (C) Diabetic peripheral vascular disease.

**A**


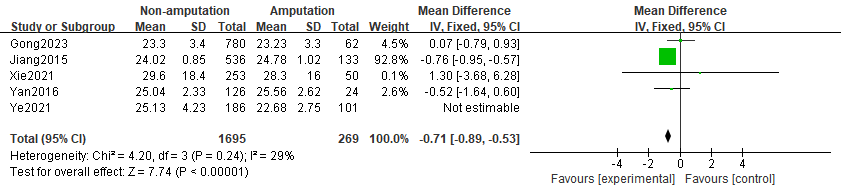


**B**


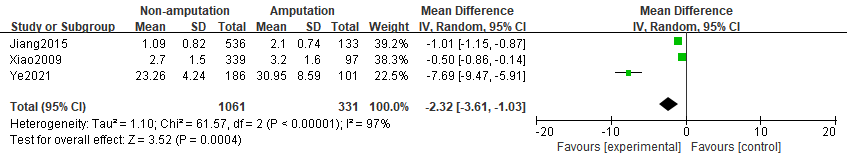


**C**


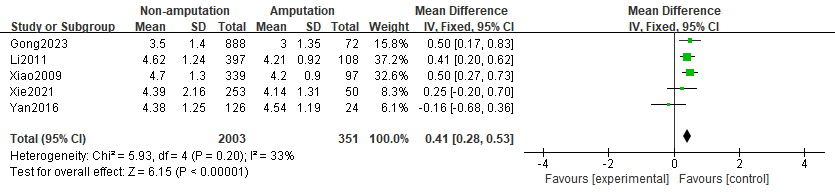


**Supplementary material 4**. Comparison of foot-related characteristics among the non-amputation and amputation groups. (A) BMI; (B) Duration of DFUs; (C) TC.

**A**


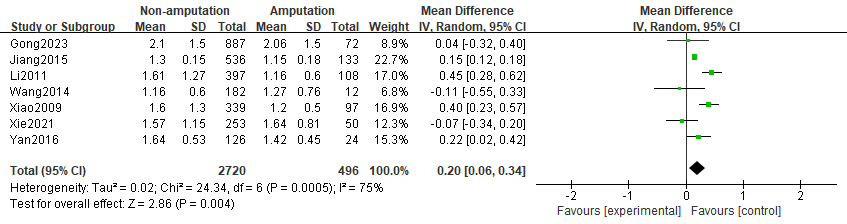


**B**


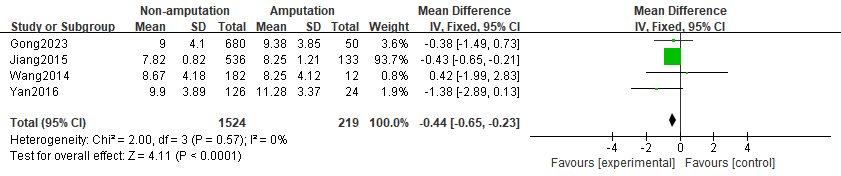


**C**


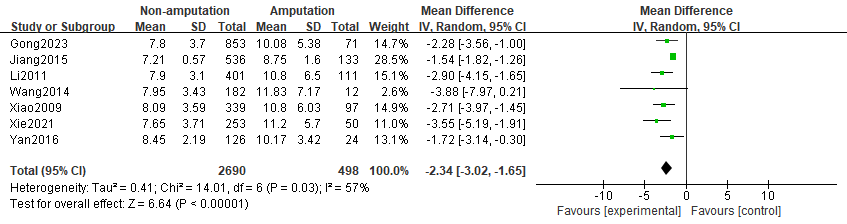


**Supplementary material 5**. Comparison of foot-related characteristics among the non-amputation and amputation groups. (A) TG; (B) FBG; (C) WBC.

**A**


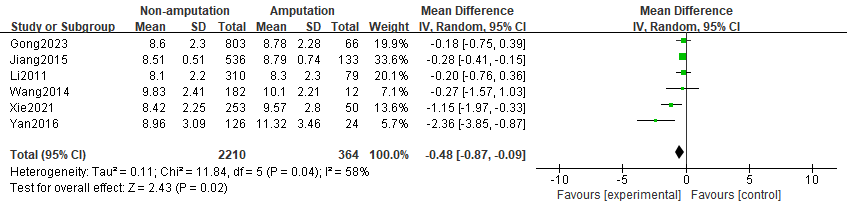


**B**


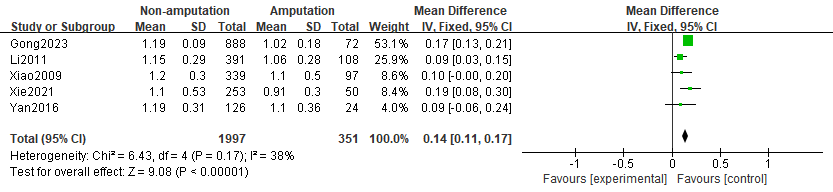


**C**


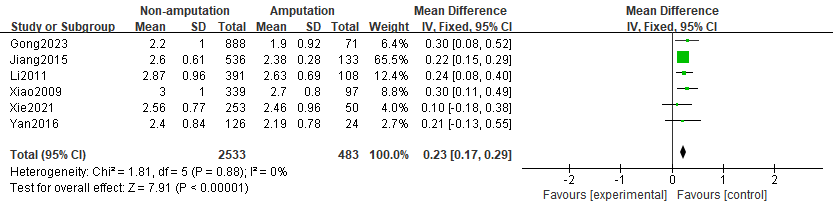


**Supplementary material 6**. Comparison of foot-related characteristics among the non-amputation and amputation groups. (A) HbA1c; (B) HDL-C; (C) LDL-C.

**A**


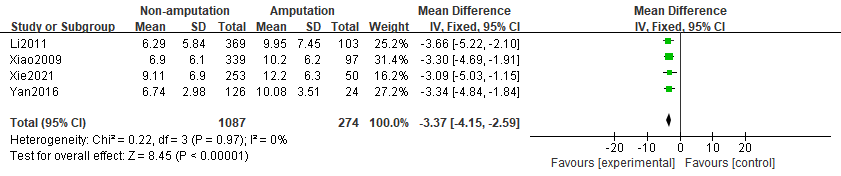


**B**


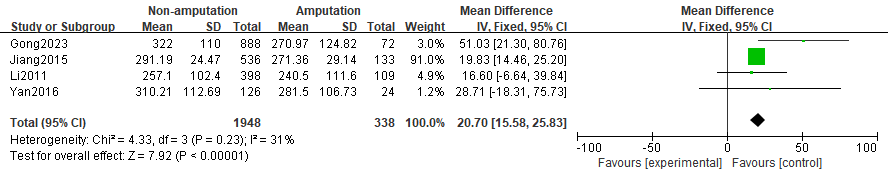


**Supplementary material 7**. Comparison of foot-related characteristics among the non-amputation and amputation groups. (A) hs-CRP; (B) Ua.

**Supplementary material 8. PRISMA_2020_checklist**

| **Section and Topic** | **Item #** | **Checklist item** | **Location where item is reported** |
| --- | --- | --- | --- |
| **TITLE** | | |  |
| Title | 1 | Identify the report as a systematic review. | See the Title. |
| **ABSTRACT** | | |  |
| Abstract | 2 | See the PRISMA 2020 for Abstracts checklist. | See the Abstract. |
| **INTRODUCTION** | | |  |
| Rationale | 3 | Describe the rationale for the review in the context of existing knowledge. | See the Introduction，the Paragraph 6. |
| Objectives | 4 | Provide an explicit statement of the objective(s) or question(s) the review addresses. | See the Introduction，the Paragraph 6. |
| **METHODS** | | |  |
| Eligibility criteria | 5 | Specify the inclusion and exclusion criteria for the review and how studies were grouped for the syntheses. | See the Methods, “Search strategy”. |
| Information sources | 6 | Specify all databases, registers, websites, organisations, reference lists and other sources searched or consulted to identify studies. Specify the date when each source was last searched or consulted. | See the Methods, “Study selection”. |
| Search strategy | 7 | Present the full search strategies for all databases, registers and websites, including any filters and limits used. | See the Methods, “Study selection”. |
| Selection process | 8 | Specify the methods used to decide whether a study met the inclusion criteria of the review, including how many reviewers screened each record and each report retrieved, whether they worked independently, and if applicable, details of automation tools used in the process. | See the Methods, “Data extraction and quality assessment”. |
| Data collection process | 9 | Specify the methods used to collect data from reports, including how many reviewers collected data from each report, whether they worked independently, any processes for obtaining or confirming data from study investigators, and if applicable, details of automation tools used in the process. | See the Methods, “Data extraction and quality assessment”. |
| Data items | 10a | List and define all outcomes for which data were sought. Specify whether all results that were compatible with each outcome domain in each study were sought (e.g. for all measures, time points, analyses), and if not, the methods used to decide which results to collect. | See the Methods, “Data extraction and quality assessment”. |
|  | 10b | List and define all other variables for which data were sought (e.g. participant and intervention characteristics, funding sources). Describe any assumptions made about any missing or unclear information. | See the Methods, “Data extraction and quality assessment”. |
| Study risk of bias assessment | 11 | Specify the methods used to assess risk of bias in the included studies, including details of the tool(s) used, how many reviewers assessed each study and whether they worked independently, and if applicable, details of automation tools used in the process. | See the Methods, “Data extraction and quality assessment”. |
| Effect measures | 12 | Specify for each outcome the effect measure(s) (e.g. risk ratio, mean difference) used in the synthesis or presentation of results. | See the Methods, “Statistical analysis”. |
| Synthesis methods | 13a | Describe the processes used to decide which studies were eligible for each synthesis (e.g. tabulating the study intervention characteristics and comparing against the planned groups for each synthesis (item #5)). | See the Methods, “Statistical analysis”. |
|  | 13b | Describe any methods required to prepare the data for presentation or synthesis, such as handling of missing summary statistics, or data conversions. | See the Methods, “Statistical analysis”. |
|  | 13c | Describe any methods used to tabulate or visually display results of individual studies and syntheses. | See the Methods, “Statistical analysis”. |
|  | 13d | Describe any methods used to synthesize results and provide a rationale for the choice(s). If meta-analysis was performed, describe the model(s), method(s) to identify the presence and extent of statistical heterogeneity, and software package(s) used. | See the Methods, “Statistical analysis”. |
|  | 13e | Describe any methods used to explore possible causes of heterogeneity among study results (e.g. subgroup analysis, meta-regression). | See the Methods, “Statistical analysis”. |
|  | 13f | Describe any sensitivity analyses conducted to assess robustness of the synthesized results. | See the Methods, “Statistical analysis”. |
| Reporting bias assessment | 14 | Describe any methods used to assess risk of bias due to missing results in a synthesis (arising from reporting biases). | See the Methods, “Statistical analysis”. |
| Certainty assessment | 15 | Describe any methods used to assess certainty (or confidence) in the body of evidence for an outcome. | See the Methods, “Statistical analysis”. |
| **RESULTS** | | |  |
| Study selection | 16a | Describe the results of the search and selection process, from the number of records identified in the search to the number of studies included in the review, ideally using a flow diagram. | See the Results, “Selection of studies and basic characteristics”. |
|  | 16b | Cite studies that might appear to meet the inclusion criteria, but which were excluded, and explain why they were excluded. | See the Results, “Selection of studies and basic characteristics”. |
| Study characteristics | 17 | Cite each included study and present its characteristics. | See the Results, “Selection of studies and basic characteristics”. |
| Risk of bias in studies | 18 | Present assessments of risk of bias for each included study. | See the Results, “Quality assessment”. |
| Results of individual studies | 19 | For all outcomes, present, for each study: (a) summary statistics for each group (where appropriate) and (b) an effect estimate and its precision (e.g. confidence/credible interval), ideally using structured tables or plots. | See the Results, “Selection of studies and basic characteristics”. |
| Results of syntheses | 20a | For each synthesis, briefly summarise the characteristics and risk of bias among contributing studies. | See the Results, “Incidence of amputation in Chinese patients with DFUs”. |
|  | 20b | Present results of all statistical syntheses conducted. If meta-analysis was done, present for each the summary estimate and its precision (e.g. confidence/credible interval) and measures of statistical heterogeneity. If comparing groups, describe the direction of the effect. | See the Results, “Subgroup analysis”. |
|  | 20c | Present results of all investigations of possible causes of heterogeneity among study results. | See the Results, “Subgroup analysis”, “Publication bias”, “Sensitivity analysis”. |
|  | 20d | Present results of all sensitivity analyses conducted to assess the robustness of the synthesized results. | See the Results, “Sensitivity analysis”. |
| Reporting biases | 21 | Present assessments of risk of bias due to missing results (arising from reporting biases) for each synthesis assessed. | See the Results, “Publication bias”. |
| Certainty of evidence | 22 | Present assessments of certainty (or confidence) in the body of evidence for each outcome assessed. | See the Results, “Subgroup analysis”, “Publication bias”, “Sensitivity analysis”. |
| **DISCUSSION** | | |  |
| Discussion | 23a | Provide a general interpretation of the results in the context of other evidence. | See the “Discussion”, Paragraph 1-6. |
|  | 23b | Discuss any limitations of the evidence included in the review. | See the “Discussion”, Paragraph 7. |
|  | 23c | Discuss any limitations of the review processes used. | See the “Discussion”, Paragraph 7. |
|  | 23d | Discuss implications of the results for practice, policy, and future research. | See the “Discussion”, Paragraph 8. |
| **OTHER INFORMATION** | | |  |
| Registration and protocol | 24a | Provide registration information for the review, including register name and registration number, or state that the review was not registered. | See the Method, “Protocol”. |
|  | 24b | Indicate where the review protocol can be accessed, or state that a protocol was not prepared. | See the Method, “Protocol”. |
|  | 24c | Describe and explain any amendments to information provided at registration or in the protocol. | See the Method, “Protocol”. |
| Support | 25 | Describe sources of financial or non-financial support for the review, and the role of the funders or sponsors in the review. | See the “Funding”. |
| Competing interests | 26 | Declare any competing interests of review authors. | See the “Declaration of conflict of interest”. |
| Availability of data, code and other materials | 27 | Report which of the following are publicly available and where they can be found: template data collection forms; data extracted from included studies; data used for all analyses; analytic code; any other materials used in the review. | All the data can be found in the Supplementary materials, which has been mentioned in the manuscript. |

*From:*  Page MJ, McKenzie JE, Bossuyt PM, et al. The PRISMA 2020 statement: an updated guideline for reporting systematic reviews. BMJ. 2021;372:n71. doi: 10.1136/bmj.n71 For more information, visit: <http://www.prisma-statement.org/>A
